# Correlation analysis of serum SERPINA3, CLEC2 and hs-CRP/ALB with major adverse cardiovascular events (MACE) after percutaneous coronary intervention (PCI) in STEMI

**DOI:** 10.5937/jomb0-62259

**Published:** 2026-06-16

**Authors:** Yuncong Ma, Suxia Fang, Shasha Liu, Zhencong Jiang

**Affiliations:** 1 Zhejiang University School of Medicine, Department of Cardiology, The Second Affiliated Hospital, Hangzhou City, China; 2 The First Affiliated Hospital of Henan University of Chinese Medicine, Cardiovascular Research Centre, Zhengzhou City, China

**Keywords:** acute ST-segment elevation myocardial infarction, percutaneous coronary intervention, members of the Ctype lectin domain family 2, serine protease inhibitor family member A3, hypersensitive C-reactive protein/albumin, akutni infarkt miokarda sa elevacijom ST segmenta, perkutana koronarna intervencija, C-lezitin domenska porodica 2, serinski proteaza inhibitor familije A3, visoko osetljivi C-reaktivni protein/albumin

## Abstract

**Background:**

To examine the main adverse cardiovascular events that occur in individuals who have had an acute ST-segment elevation myocardial infarction (STEMI) after receiving percutaneous coronary intervention (PCI), such as serum C-type lectin domain family member 2 (CLEC2), serine protease inhibitor family member A3 (SERPINA3), and high-sensitivity C-reactive protein/albumin (hs-CRP/ALB) levels.

**Methods:**

The STEMI group included 132 patients who were observed for 1 year after hospitalisation between January 2023 and September 2024. The patients were divided into two groups: the MACE group and the non-MACE group, based on whether MACE came after PCI. Additionally, the control group consisted of 68 healthy individuals who were physically examined at the hospital during the same time period. Using an enzyme-linked immunosorbent test, the levels of serum CLEC2, SERPINA3, hs-CRP and ALB were measured in each research participant, and the hs-CRP to ALB ratio was computed. After PCI, multivariate logistic regression was used to examine variables associated with MACEs in STEMI patients. Receiver operating characteristic (ROC) curves were used to analyse the predictive value of single and combined detection of serum CLEC2, SERPINA3, and hs-CRP/ALB for MACEs in STEMI patients after PCI.

**Results:**

The incidence of MACE after PCI in 132 STEMI patients was 31.06% (41/132). The levels of serum CLEC2, SERPINA3 and hs-CRP/ALB in the STEMI group were significantly greater than those in the control group (P&lt; 0.05). Independent risk factors for MACEs following PCI in STEMI patients included age &gt;62 years, Killip grade &gt; grade III, cardiac troponin I level &gt;1.7 ng/mL, CLEC2 level &gt;155 pg/mL, SERPINA3 level &gt;350 ng/L, and hs-CRP/ALB &gt;0.50 (P&lt; 0.05), while left ventricular ejection fraction &gt;50% was an independent protective factor. The area under the ROC curve (0.856) of the combined detection of serum CLEC2, SERPINA3, and hs-CRP/ALB for the prediction of MACEs in STEMI patients after PCI was greater than that of the individual detection of each index.

**Conclusions:**

Serum CLEC2 levels &gt;155 pg/mL, SERPINA3 levels &gt;350 ng/L, and hs-CRP/ALB&gt;0.50 are closely related to the occurrence of MACEs in STEMI patients after PCI and can be used as auxiliary predictive indicators for the occurrence of MACEs in STEMI patients after PCI.

## Abstract (sr)

**Uvod:**

Cilj je bio da se ispitaju glavni nepovoljni kardio-vaskularni događaji (MACE) kod pacijenata sa akutnim infarktom miokarda sa elevacijom ST segmenta (STEMI) nakon perkutane koronarne intervencije (PCI), sa posebnim osvrtom na ulogu serumske C-lezitin domenske porodice 2 (CLEC2), serinskog proteaza inhibitora A3 (SERPINA3) i odnosa visoko osetljivog C-reaktivnog proteina i albumina (hs-CRP/ALB).

**Metode:**

U studiju je uključeno 132 pacijenta sa STEMI koji su primili PCI u periodu od januara 2023. do septembra 2024. godine, a praćeni su godinu dana nakon otpusta iz bolnice. Na osnovu pojave MACE nakon PCI, pacijenti su podeljeni u MACE i ne-MACE grupu. Kontrolnu grupu je činilo 68 zdravih ispitanika koji su u istom periodu obavljali sistematske preglede. Serum CLEC2, SERPINA3, hs-CRP i ALB određeni su pomoću ELISA testa, a izračunat je i odnos hs-CRP/ALB. Multivarijantna logistička regresija korišćena je za identifikaciju nezavisnih faktora rizika za MACE, dok su ROC krive korišćene za procenu prediktivne vrednosti pojedinačnog i kombinovanog određivanja CLEC2, SERPINA3 i hs-CRP/ALB.

**Rezultati:**

Incidenca MACE nakon PCI kod pacijenata sa STEMI bila je 31,06% (41/132). Nivoi seruma CLEC2, SERPINA3 i hs-CRP/ALB bili su značajno viši u STEMI grupi u poređenju sa kontrolnom grupom (P&lt; 0,05). Nezavisni faktori rizika za MACE nakon PCI uključivali su starost &gt;62 godine, Killip klas &gt;III, nivo troponina I &gt;1,7 ng/mL, CLEC2 &gt;155 pg/mL, SERPINA3 &gt;350 ng/L i hs-CRP/ALB &gt;0,50 (svi P&lt; 0,05), dok j'e frakcij'a izbacivanj'a leve komore &gt;50% predstavljao nezavisni zaštitni faktor. Kombinovano određivanj'e CLEC2, SERPINA3 i hs-CRP/ALB j'e dalo vecu površinu ispod ROC krive (AUC= 0,856) u odnosu na pojedinačne indikatore.

**Zaključak:**

Povišeni nivoi seruma CLEC2 (&gt;155 pg/mL), SERPINA3 (&gt;350 ng/L) i hs-CRP/ALB (&gt;0,50) povezani su sa pojavom MACE kod pacijenata sa STEMI nakon PCI i mogu se koristiti kao dodatni prediktivni biomarkeri. Potrebne su dalje studije velikog uzorka i multi-centrične studije radi potvrde ovih nalaza.

## Introduction

The most severe form of coronary heart disease and the primary cause of patient mortality and disability is acute ST-segment elevation myocardial infarction (STEMI) [1]. Percutaneous coronary intervention (PCI) is the most effective treatment for opening occluded vessels in STEMI patients. Therefore, it is crucial to predict MACEs after PCI at an early stage. Studies [2][3][4] have shown that thrombus formation following rupture of atherosclerotic (AS) plaques is the mechanism of STEMI. Platelet activation and inflammatory responses contribute to the pathogenesis of AS. C-type lectin domain family member 2 (CLEC2) is a transmembrane receptor that induces platelet activation through interaction with its endogenous ligand, flatfoot protein [5]. Serine protease inhibitor family member A3 (SERPINA3) is a protease inhibitor that helps regulate various biological processes, including inflammatory responses, complement activation, and cell migration. The inflammatory response status can be accurately reflected by a novel measure of inflammation, the hypersensitive C-reactive protein/albumin (hs-CRP/ALB) ratio [6][7][8].

The occurrence of major adverse cardiovascular events (MACEs) in patients with acute ST-segment elevation myocardial infarction (STEMI) after percutaneous coronary intervention (PCI) remains a significant clinical challenge. It seriously affects patient prognosis [9]. Although PCI can rapidly open culprit vessels, the persistent inflammatory response, endothelial dysfunction and tendency toward thrombosis after surgery are the core pathophysiological mechanisms driving the progression of atherosclerosis, stent thrombosis and other MACEs [10]. Therefore, there is an urgent need to identify early, sensitive, and effective biological markers to accurately identify patients at high risk of postoperative MACE, optimise clinical intervention strategies, and improve patient outcomes [11][12][13]. At present, the commonly used risk stratification tools and some single biochemical indicators (such as hs-CRP) have certain limitations in predicting MACEs after PCI. Serum α 1-antitrypsin (SERPINA3) is an acute-phase reactive protein and serine protease inhibitor, and C-type lectin-like receptor 2 (CLEC2) is an essential receptor for platelet activation [14][15].

To explore the correlation between serum levels of SERPINA3, CLEC2, and hs-CRP/ALB, and their combinations, and the occurrence of MACEs after PCI, we aimed to provide a more comprehensive new perspective for clinical risk assessment. It has significant theoretical significance and clinical application prospects for achieving individualised risk stratification. Ultimately, early detection and targeted intervention will reduce the prevalence of MACE and improve patients' quality of life.

## Materials and methods

### General information

The STEMI group comprised 132 patients with STEMI admitted to our hospital between January 2023 and September 2024; 86 were male and 46 were female. With an average age of 61.23±9.25 years, the ages varied from 39 to 82 years. The body mass index ranged from 18.68 to 28.29 kg/m^2^, with an average of 23.41±2.05 kg/m^2^. Criminal vessels: Forty-five cases involved the left anterior descending branch, 20 cases involved the left circumflex branch, and 67 cases involved the right coronary artery. Killip classification: 104 patients were Grade I to II, and 28 were Grade III to IV Underlying diseases included 72 cases of hypertension, 34 cases of diabetes, 44 cases of hyperlipidemia, 16 cases of chronic kidney disease, 21 cases of chronic liver disease, and 14 cases of chronic obstructive pulmonary disease. Sixty-six patients smoked.

They were separated into two groups based on whether or not MACE occurred: the MACE group and the non-MACE group. An additional 68 healthy people, 24 female and 44 male, were selected as the control group for physical tests at our hospital during that time. The ages ranged from 27 to 76 years, with an average of 60.57±5.36 years. The body mass index ranged from 18.77 to 26.36 kg/m^2^, with an average of 23.17±1.83 kg/m^2^.

### Inclusion and exclusion criteria

Inclusion criteria: (1) Meeting the STEMI diagnostic criteria in the »Guidelines for the Diagnosis and Treatment of Acute ST-segment Elevation Myocardial Infarction (2022)«; (2) The time from the onset of chest pain to admission is less than 12 hours; (3) Have indications for PCI treatment; (4) Be able to accept visitors.

Exclusion criteria: (1) Age <18 years old; (2) Have a history of thromboembolic diseases; (3) Damage to the hematopoietic and immune systems, as well as severe liver and kidney dysfunction, etc. (4) Combined malignant tumors, as well as valve diseases, ventricular septal defects, atrial septal diseases and other structural heart diseases; (5) Has received hormone replacement therapy in the past; (6) There is a history of blood transfusion or the use of steroid hormones, non-steroidal anti-inflammatory drugs, anticoagulant drugs, contraceptive drugs, or estrogen within the last 3 months; (7) Incomplete medical records; (8) Death within the hospital.

### Detection of serum CLEC2, SERPINA3, hs-CRP and ALB

After serum separation, CLEC2 (kit purchased from Shanghai Baililai Biotechnology Co., Ltd., number: BLL 104814E) and SERPINA3 (kit purchased from Shanghai Yuduo Biotechnology Co., LTD., number:) were detected via enzyme-linked immunosorbent assay. The level of YEF20252 was detected by latex-enhanced immunoturbidimetry for hs-CRP (kit purchased from Shanghai Yaji Biotechnology Co., Ltd., number: E024), and the level of ALB was detected by bromocresol green colourimetry (kit purchased from Guangzhou Weibo Technology Co., LTD., number: A028-1-1), and the Eis-CRP/aLb ratio was calculated.

### Principles of laboratory testing

In this study, the levels of high-sensitivity C-reactive protein (hs-CRP), albumin (ALB), CLEC2, and SERPINA3 in patients with acute ST-segment elevation myocardial infarction (STEMI) after percutaneous coronary intervention (PCI) were quantified by enzyme-linked immunosorbent assay (ELISA). To evaluate its correlation with major adverse cardiovascular events (MACE).

The following biochemical indicators were measured using commercially available assay kits:

1. High-sensitivity C-reactive protein (hs-CRP) was determined with a kit provided by Chem Company (item no. 80955);
2. Albumin levels were assessed using a kit from Elabscience Company (item no. E-EL-H013);
3. CLEC2 (C-type lectin receptor 2) concentrations were measured with a BioSource Company kit (item no. MBS722493E); and
4. SERPINA3 (α1-antitrypsin 3) was detected using reagents supplied by Antibodies-Online (item no. ABIN6961964).

### Follow-up investigation

All patients were followed up for one year by phone or in the outpatient department after PCI. It was followed up on till September 2025. The prevalence of MACEs, which include stent thrombosis, cardiogenic mortality, recurrent unstable angina pectoris, revascularisation, and recurrent myocardial infarction, was statistically examined.

### Statistical analysis methods

Data analysis was conducted using SPSS 25.0. Count data were expressed as counts or percentages, and the χ^2^ test was used. Two groups were compared using a t-test, and normally distributed data are displayed as x̄±s. M(P25, P75) is the expression for non-normally distributed measurement data. The influencing factors of MACEs in STEMI patients following PCI were examined using multivariate logistic regression, and a regression risk prediction model was created by combining several indicators. The predictive value of serum CLEC2, SERPINA3 and the hs-CRP/ALB ratio for MACEs in STEMI patients after PCI was analysed via receiver operating characteristic (ROC) curves.

## Results

### Comparison of serum CLEC2 and SERPINA3 levels and the hs-CRP/ALB ratio between the STEMI group and the control group

The levels of serum CLEC2, SERPINA3 and hs-CRP/ALB in the STEMI group were significantly greater than those in the control group (P<0.05) (see Table 1).

**Table 1:** Comparison of serum CLEC2, SERPINA3 levels and hs CRP/ALB between TEMI group and control group.

Group	n	CLEC2 (pg/mL)	SERPINA3 (ng/L)	hs-CRP/ALB
STEMI group	132	154.66±23.66	332.22 (212.60,477.11)	0.47 (0.42,0.54)
Control group	68	103.75±17.96	112.15 (66.30,170.36)	0.22 (0.21,0.23)
t/Z		16.985	4.328	3.717
P		<0.001	<0.001	<0.001

The levels of serum CLEC2 and SERPINA3, and the hs-CRP/ALB ratio, were compared between patients with acute ST-segment elevation myocardial infarction (STEMI) and healthy controls. The results showed that the levels of serum CLEC2 and SERPINA3 in the STEMI group were significantly higher than those in the control group, and there was also a significant difference in the hs-CRP/ALB ratio. These differences indicate that in STEMI patients after acute myocardial infarction, the elevated levels of CLEC2 and SERPINA3 in serum and the changes in the hs-CRP/ALB ratio may be closely related to the occurrence of adverse cardiovascular events.

### Comparison of general information and levels of various laboratory indicators between the MACE group and the non-MACE group

Among 132 STEMI patients after PCI, 11 experienced recurrent myocardial infarction, 8 experienced heart failure, 9 experienced recurrent unstable angina pectoris, 6 experienced stent thrombosis, and 7 experienced cardiogenic death; the incidence of MACEs was 31.06% (41/132). The proportion of patients with a Killip grade ≥ III was higher in the MACE group than in the non-MACE group. Serum cTnI, CLEC2, SERPINA3, and hs-CRP/ALB levels were all higher in the MACE group than in the nonMACE group, and the LVEF was additionally lower. P<0.05 indicated that the differences were statistically significant (see Table 2).

**Table 2:** Comparison of general information and laboratory indicator levels between MACE and non-MACE groups.

Item	MACE group (n=41)	Non MACE group (n = 91)	X2/t/Z	P
Gender			2.865	0.091
Male	31 (75.61)	55 (60.44)		
Female	10 (24.39)	36 (39.56)		
Age (years)	64.93±8.82	59.56±8.99	3.194	0.002
Body Mass Index (kg/m^2^)	23.78±1.77	23.24±2.16	1.402	0.163
Smoking	24 (58.54)	42 (46.15)	1.734	0.188
Underlying disease				
Hypertension	25 (60.98)	47 (51.65)	0.992	0.319
Diabetes	12 (29.27)	22 (24.18)	0.383	0.536
Hyperlipidemia	16 (39.02)	28 (30.77)	0.867	0.352
Chronic kidney disease	6 (14.63)	10 (10.99)	0.093	0.760
Chronic liver disease	8 (19.51)	13 (14.29)	0.577	0.447
Chronic obstructive pulmonary disease	6 (14.63)	8 (8.79)	2.126	0.145
Criminal vascular system			1.350	0.509
Left anterior descending branch	15 (36.59)	30 (32.97)		
Left circumflex branch	4 (9.76)	16 (17.58)		
Right coronary artery	22 (53.66)	45 (49.45)		
Killip rating ≥ Level III	19 (46.34)	9 (9.89)	22.472	<0.001
Total cholesterol (mmol/L)	4.55±0.37	4.45±0.33	1.551	0.123
Triglycerides (mmol/L)	1.74±0.44	1.66±0.42	0.998	0.320
HDL-C (mmol/L)	0.96 (0.83, 1.11)	1.03 (0.92, 1.16)	1.379	0.168
LDL-C (mmol/L)	3.21±0.35	3.12±0.38	1.290	0.199
White blood cell count (×10^9^/L)	8.82±3.21	8.70±3.42	0.190	0.850
Platelet count (×10^9^/L)	177.08±20.01	176.87±18.92	0.058	0.954
cTnI (ng/mL)	1.84±0.31	1.54±0.28	5.508	<0.001
CK-MB (U/L)	43.26 (36.29, 46.58)	39.65 (35.79, 44.29)	1.589	0.112
LVEF (%)	47.04±6.18	52.80±7.91	4.127	<0.001
CLEC2 (pg/mL)	170.70±23.31	147.44±20.09	3.852	<0.001
SERPINA3 (ng/L)	444.52 (358.29, 615.35)	270.26 (172.26, 389.37)	4.613	<0.001
hs-CRP/ALB	0.55 (0.47, 0.61)	0.45 (0.40, 0.51)	2.108	0.035

Comparative analysis revealed that specific serum markers, including CLEC2, SERPINA3, and the hs-CRP/ALB ratio, are elevated in the MACE group, suggesting a strong association with MACE. Further analysis suggests that the degree of cardiac function impairment, especially the reduction in left ventricular ejection fraction, is a key factor in the occurrence of MACE.

### Multivariate logistic regression analysis of the influencing factors of MACEs after PCI in STEMI patients

An unconditional logistic regression model was established, with whether MACE occurred after PCI as the dependent variable (occurrence = 1, no = 0). The seven indicators with statistically significant differences in Table 2 are taken as independent variables. Given the small sample size, dimensionality reduction treatment of the factors themselves and the number of factors was adopted according to the statistical design: (1) Some independent variables with continuous values were transformed into binary variables based on the total mean or median of the two groups (rounded appropriately); (2) By adopting the stepwise forward method, the order of including independent variables was optimised based on multiple experimental regressions so that some indicators were excluded first. Age ≥62 years, Killip grade ≥ grade III, cTnI level ≥1.7 ng/mL, CLEC2 level ≥155 pg/mL, SERPINA3 level ≥350 ng/L, and hs-CRP/ALB ratio ≥0.50 were found to be independent risk factors for MACEs following PCI in STEMI patients (P<0.05), whereas it was found that LVEF≥50% was a separate protective factor (see Table 3).

**Table 3:** Multivariate logistic regression analysis of the influencing factors of MACE in STEMI patients after PCI.

Factor	Assignment	β	SE	Wald x^2^	P	OR (95% CI)
Constant	-	-0.103	0.046	4.996	0.02	
Age	≥ 62 years old = 1, <62 years old=0	0.164	0.077	4.524	0.033	1.178 (1.013~1.370)
Killip classification	≥ Level III=1, <Level III=0	1.190	0.509	5.463	0.019	3.286 (1.212~8.916)
cTnI	≥1.7 ng/mL=1, <1.7 ng/mL=0	0.356	0.092	15.099	<0.001	1.427 (1.193~1.708)
LVEF	≥50%=1, <50%=0	-0.14	0.058	5.773	0.016	0.869 (0.776~0.975)
CLEC2	≥155 pg/mL=1, <155 pg/mL=0	0.309	0.080	15.045	<0.001	1.362 (1.165~1.592)
SERPINA3	≥350 ng/L=1, <350 ng/L=0	0.409	0.138	8.804	0.003	1.506 (1.149~1.972)
hs-CRP/ALB	≥0.50 = 1, <0.50=0	0.202	0.088	5.220	0.022	1.224 (1.029~1.455)

### The predictive value of single and combined detection of serum CLEC2, SERPINA3, and hs-CRP/ALB for MACEs after PCI in STEMI patients

Both positive and negative samples from the MACE group and the non-MACE group were used in the ROC curve study. When the CLEC2, SERPINA3 and hs-CRP/ALB indicators are combined and applied, a logistic regression risk prediction model is established.

**Figure 1:**
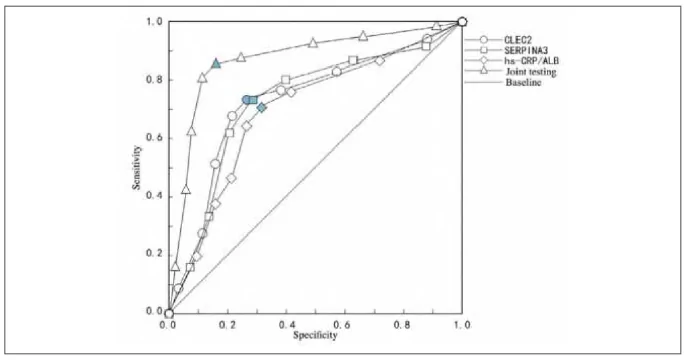
ROC curves of serum CLEC2, SERPINA3, and hs-CRP/ALB single and combined detection for MACE in STEMI patients after PCI.

Taking Logit (P) = -0.033 + 0.063 X XCLEC2 + 0.027 X XSERPINA3 + 1.145 X Xhs-CRP/ALB as the virtual probability index for the combined application, the ROC curve was then constructed. The areas under the curve (AUCs) of serum CLEC2, SERPINA3, and hs-CRP/ALB for individual and combined detection were 0.756 (95% CI: 0.544-0.950), 0.742 (95% CI: 0.543-0.921), 0.720 (95% CI: 0.488-0.9), and 0.856 (95% CI: 0.727-0.965), respectively (see Table 4 and Figure 1).

**Table 4:** The predictive value of serum CLEC2, SERPINA3, hs CRP/ALB single and combined detection for MACE after PCI in STEMI patients.

Indicator	AUC (95% CI)	Optimal truncation<br>value	Sensitivity	Specificity	Yoden Index	Accuracy
CLEC2	0.756 (0.544 0.950)	160 pg/mL	0.732	0.736	0.468	0.735
SERPINA3	0.742 (0.543 0.921)	350 ng/L	0.732	0.714	0.446	0.720
hs-CRP/ALB	0.720 (0.488 0.925)	0.50	0.707	0.681	0.388	0.689
3 Joint Proj'ects	0.856 (0.727 0.965)	-	0.854	0.835	0.689	0.841

## Discussion

STEMI is an acute ischemic necrosis of the myocardium caused by secondary thrombosis based on rupture, erosion, and endothelial injury of unstable coronary artery plaques [16][17][18]. Comparing myocardial infarction that is not ST-segment elevation, the infarction area and degree of vascular stenosis associated with STEMI are often more severe, and the risk of disability and death is extremely high worldwide [19]. Although the risk of disability and death in STEMI patients has significantly decreased in recent years with improvements in PCI technology and equipment, there is still a relatively high probability of MACEs after PCI, which is an important reason for the decline in the quality of life of STEMI patients [20]. Early MACE risk prediction in STEMI patients following PCI is helpful for guiding early risk stratification and action and for enhancing patient outcomes. Acute occlusive endovascular thrombosis caused by the rupture or erosion of AS plaques is the fundamental mechanism underlying the occurrence and progression of STEMI. Studies have shown that when platelets are activated, they can secrete various inflammatory mediators to regulate cell chemotaxis, aggregation and adhesion, causing multiple cells to accumulate at the injury site and interweave to form thrombi, promoting the occurrence and development of AS [21][22][23]. CLEC2 is a calcium-dependent lectin receptor that is expressed mainly on the surface of platelets and megakaryocytes [24]. It can form dimers by binding endogenous ligands, planoproteins, and exogenous ligands to snake venom protein via its long circular domain, thereby promoting highly ordered aggregation of CLEC2 on platelet surfaces and causing platelet aggregation and activation. Given CLEC2's significant role in platelet activation, multiple studies have reported a relationship between CLEC2 and AS: Vascular smooth muscle cells can stimulate platelets via CLEC2, leading to erosion of AS plaques and thrombosis [25]. In a mouse model of AS susceptibility induced by carotid artery ligation, upregulation of CLEC2 expression promoted platelet accumulation under the endothelium to form thrombi, whereas, conversely, it reduced platelet aggregation and inhibited the progression of AS, indicating that CLEC2 is closely associated with AS [26]. Moreover, some scholars have noted that elevated plasma CLEC2 expression is independently associated with CHD. This finding is due to CLEC2's involvement in AS and thrombosis [27]. The higher the serum CLEC2 level is, the greater the thrombotic burden in STEMI patients. Therefore, the risk of MACEs after PCI is greater. A high thrombotic burden can lead to distal embolism after PCI, reduce myocardial blood perfusion and the number of surviving myocardia, and increase the risk of MACEs in STEMI patients [28].

Vascular endothelial dysfunction is the initiating factor in the development of AS, and the inflammatory response occurs throughout the entire process. Cathepsin is a type of intracellular peptide bond hydrolase that can participate in the inflammatory response process by modifying cytokines, chemokines, and cell surface receptors [29]. SERPINA3 (also known as α 1-antichymotrypsin) is the first acute-phase plasma proteolytic enzyme inhibitor discovered and is released when cathepsin is activated. The endothelium and smooth muscle cells of AS animals exhibit elevated SERPINA3 expression, suggesting a potential link between SERPINA3 and AS development. Elevated blood SERPINA3 levels have been linked in recent years to the worsening of heart failure and a higher patient death rate, indicating that myocardial injury may also be a factor [30]. The analysis indicated that, as an acute-phase response protein, elevated SERPINA3 levels reflected an aggravated inflammatory response, which, in turn, increased myocardial injury [31]. Inflammatory responses can lead to heart failure and cardiogenic death by causing myocardial fibrosis, increasing the risk of MACEs. SERPINA3 is highly expressed in infarcted hearts and can activate the important inflammatory response signalling pathway, the nuclear receptor NR4A1 [32].

hs-CRP is an acute-phase protein secreted by the liver. When inflammatory responses stimulate body tissues and cells, they can be rapidly synthesised and released into the bloodstream, serving as markers of these responses [33]. ALB is a negative time-phase reaction protein secreted by the liver that has functions such as maintaining osmotic pressure and providing nutrition. When inflammatory responses stimulate the body's tissues and cells, liver synthesis can be inhibited. Since hs-CRP levels can also increase in infectious diseases, ALB levels are also affected by factors such as appetite, metabolism, and infection [34]. Therefore, the comprehensive characteristics of the hs-CRP/ALB indicators can better reflect the inflammatory response status. Studies [35][36][37] have shown that hs-CRP/ALB can be used as a prognostic marker for conditions such as sepsis complicated by cardiac dysfunction and chronic obstructive pulmonary disease. However, at present, reports in the literature on the occurrence of MACEs after PCI in STEMI patients with high-CRP/ALB are relatively rare. The serum hs-CRP/ALB ratio is elevated in STEMI patients. The inflammatory response in STEMI patients increases hs-CRP levels and decreases ALB levels, thereby elevating the hs-CRP/ALB ratio. The reason for this finding was that the higher the hs-CRP/ALB, the more severe the inflammatory response in STEMI patients, which could increase the risk of MACEs by aggravating the thrombotic burden and impairing cardiac function. The results of this study also revealed that age ≥62 years, Killip grade ≥III, cTnI ≥1.7 ng/mL, and LVEF ≥50% are independent predictors of MACEs in STEMI patients after PCI. The reason is that as one gets older, the condition of the coronary arteries worsens. Therefore, the risk of MACEs after PCI is greater. The higher the Killip classification, the higher the cTnI level, and the lower the LVEF, the poorer the cardiac function in STEMI patients. Reduced cardiac function is more likely to lead to heart failure or even sudden death. Finally, this study evaluated the predictive value of serum CLEC2, SERPINA3, and hs-CRP/ALB for MACE in STEMI patients after PCI through ROC curve analysis. The AUC for the combined detection of the three indicators for predicting MACEs in STEMI patients after PCI was higher than that for the individual detection of each indicator. These findings indicate that serum CLEC2, SERPINA3, and the hs-CRP/ALB ratio may all be auxiliary predictive indicators for MACEs after PCI in STEMI patients. The combined detection of serum CLEC2, SERPINA3, and hs-CRP/ALB by these three indicators can further improve predictive efficiency and help with early risk warning and intervention.

## Conclusion

In STEMI patients, serum CLEC2 levels ≥155 pg/mL, SERPINA3 levels 2350 ng/L, and hs-CRP/ALB ratios ≥0.50 are independent risk factors for MACEs during PCI. They may serve as supplementary predictive markers for subsequent MACEs. The combined predictive value of these three indicators is also higher. However, the findings of this study require further validation through large-sample, multi-centre research to confirm their clinical applicability and predictive reliability.

## Dodatak

### Funding

None.

### Ethical approval

This study was approved by the Medical Research Ethics Committee of our hospital [HKYS-2025-A0225].

### Conflict of interest statement

All the authors declare that they have no conflict of interest in this work.
